# Dermal Delivery of a SARS-CoV-2 Subunit Vaccine Induces Immunogenicity against Variants of Concern

**DOI:** 10.3390/vaccines10040578

**Published:** 2022-04-08

**Authors:** Christopher L. D. McMillan, Armira Azuar, Jovin J. Y. Choo, Naphak Modhiran, Alberto A. Amarilla, Ariel Isaacs, Kate E. Honeyman, Stacey T. M. Cheung, Benjamin Liang, Maria J. Wurm, Paco Pino, Joeri Kint, Germain J. P. Fernando, Michael J. Landsberg, Alexander A. Khromykh, Jody Hobson-Peters, Daniel Watterson, Paul R. Young, David A. Muller

**Affiliations:** 1 School of Chemistry and Molecular Biosciences, The University of Queensland, Brisbane, QLD 4072, Australia; c.mcmillan1@uq.edu.au (C.L.D.M.); a.azuar@uq.edu.au (A.A.); j.choo@uq.edu.au (J.J.Y.C.); n.modhiran@uq.edu.au (N.M.); a.amarillaortiz@uq.edu.au (A.A.A.); ariel.isaacs@uq.net.au (A.I.); k.honeyman@uq.net.au (K.E.H.); s.cheung@uq.edu.au (S.T.M.C.); benjamin.liang@uq.edu.au (B.L.); gfernando@vaxxas.com (G.J.P.F.); m.landsberg@uq.edu.au (M.J.L.); alexander.khromykh@uq.edu.au (A.A.K.); j.peters2@uq.edu.au (J.H.-P.); d.watterson@uq.edu.au (D.W.); p.young@uq.edu.au (P.R.Y.); 2 ExcellGene SA, CH1870 Monthey, Switzerland; maria.wurm@excellgene.com (M.J.W.); paco.pino@excellgene.com (P.P.); joeri.kint@excellgene.com (J.K.); 3 Translational Research Institute, Vaxxas Pty Ltd., Brisbane, QLD 4102, Australia; 4 Australian Infectious Diseases Research Centre, Global Virus Network Centre of Excellence, Brisbane, QLD 4072 and 4029, Australia

**Keywords:** COVID-19, SARS-CoV-2 vaccine, S protein, HexaPro, HD-MAP, cutaneous, intradermal, antibodies, virus neutralization microneedle patch

## Abstract

The ongoing coronavirus disease 2019 (COVID-19) pandemic continues to disrupt essential health services in 90 percent of countries today. The spike (S) protein found on the surface of the causative agent, the SARS-CoV-2 virus, has been the prime target for current vaccine research since antibodies directed against the S protein were found to neutralize the virus. However, as new variants emerge, mutations within the spike protein have given rise to potential immune evasion of the response generated by the current generation of SARS-CoV-2 vaccines. In this study, a modified, HexaPro S protein subunit vaccine, delivered using a needle-free high-density microarray patch (HD-MAP), was investigated for its immunogenicity and virus-neutralizing abilities. Mice given two doses of the vaccine candidate generated potent antibody responses capable of neutralizing the parental SARS-CoV-2 virus as well as the variants of concern, Alpha and Delta. These results demonstrate that this alternative vaccination strategy has the potential to mitigate the effect of emerging viral variants.

## 1. Introduction

Severe acute respiratory syndrome coronavirus 2 (SARS-CoV-2) has been rapidly spreading internationally since its emergence in December of 2019 [1,2,3,4]. The coronavirus disease 2019 (COVID-19) pandemic, caused by SARS-CoV-2, was officially classified as a pandemic by the World Health Organization (WHO) in March of 2020, as the number of cases drastically escalated worldwide [5]. Upon successful isolation of the virus from infected patients [6,7,8,9], this novel zoonotic coronavirus was identified as belonging to the *Coronaviridae* family [10]. SARS-CoV-2 is an enveloped virus and contains a positive-strand RNA genome ~30 kb in size. The genome encodes for four structural proteins: the spike (S), envelope (E), membrane (M), and nucleocapsid (N) proteins [11], which combine to form the virus particle [12,13]. Among these proteins, the S protein is an attractive target for vaccine development as it plays a crucial role in receptor binding and virus entry [14,15,16,17]. To facilitate entry into the host cells, the attachment of SARS-CoV-2 is mediated by the densely glycosylated S proteins embedded on the virion surface [18]. The receptor-binding domain (RBD; component of the S1 subunit) of the S protein targets the host cell receptor angiotensin-converting enzyme 2 (ACE2) [19]. The act of receptor engagement triggers the virus–host membrane fusion cascade resulting in viral entry. Vaccine candidates that target the surface-exposed S protein induce neutralizing antibodies that block the binding of the virus to the host cell receptor and therefore prevent infection [15].

The first generation of licensed vaccines developed by BioNTech/Pfizer, Moderna, Johnson & Johnson, and AstraZeneca target the immune response against the S protein. [20,21]. Despite the potent humoral and cellular immune responses elicited by mRNA vaccines, they require ultra-low temperatures (between −20 to −70 °C) for storage, complicating vaccine distribution in low- to middle-income countries [15,22]. Protein-based vaccines have been observed to have low-cost manufacturing procedures and stability outside of the cold chain, which will assist with COVID-19 vaccine distribution. Along with its high safety profile, protein subunits serve as a great candidate for vaccine against SARS-CoV-2. These protein subunit vaccination strategies have also been adopted by Clover Biopharmaceuticals [23], Novavax [24], Sanofi/GlaxoSmithKline [25], and SK Bioscience [26], and are now undergoing late-stage clinical evaluation [27].

All SARS-CoV-2 vaccines currently licensed are administered by needle-based injections [28,29,30,31,32,33,34,35], which imposes certain limitations on worldwide dissemination, particularly in developing countries with insufficient healthcare infrastructure and skilled workers [36,37]. As an alternative, needle-free, skin-based vaccination routes offer the possibility of a rapid distribution of vaccines with improved thermostability and simplified storage and transportation [36,37]. High-density microarray patches (HD-MAPs) have garnered much interest in vaccine delivery due to the direct delivery of the vaccine to the epidermal and higher dermal layers of the skin, which are densely packed with antigen-presenting cells [37,38,39,40]. They have demonstrated increased immunogenicity and stability outside of the cold chain in preclinical and clinical studies, both of which are extremely desirable traits for vaccines against emerging viral pathogens [41,42]. The HD-MAP used in this study is a 1 × 1 cm^2^ solid polymer array with 5000 projections of 250 µm length per cm^2^ [41]. The vaccine is coated onto microprojections using a nitrogen jet-based drying method [41] before being applied to the skin using a spring-loaded applicator at a velocity of 18–20 m/s.

Herein, we evaluated a commercially available (ExcellGene, Monthey, Switzerland), modified version of the HexaPro S protein (containing six stabilizing proline substitutions [43,44,45,46]), as an alternative vaccine antigen against SARS-CoV-2. Dose match studies were performed comparing the immune response of HD-MAP- and intradermal (i.d.)-delivered HexaPro S protein. Mice that received two doses of HexaPro S protein produced potent neutralizing antibody responses against the wildtype/ancestral SARS-CoV-2 and the highly contagious Alpha and Delta variants.

## 2. Materials and Methods

### 2.1. SARS CoV-2 HexaPro S Protein

Trimeric SARS-CoV-2 HexaPro S protein containing six stabilizing proline substitutions [43] produced by ExcellGene ( Monthey, Switzerland) was used as a vaccine antigen. The protein was produced from a stable Chinese Hamster Ovary (CHO) cell line in Bioreactors, as described previously [44]. Trimeric HexaPro S protein from SARS-CoV-2 variants of concern (Alpha (B.1.1.7; ∆H69, ∆V70, ∆Y144, N501Y, A570D, D614G, P681H, T716I, S982A, and D1118H), and Delta (B.1.617.2; T19R, G142D, ∆156E, ∆157F, R158G, L452R, T478K, D614G, P681R, and D950N)), which also contained the stabilizing HexaPro substitutions, were synthesized as reported previously [42].

### 2.2. SARS CoV-2 HexaPro S Protein Characterization

The biophysical characterization of SARS-CoV-2 HexaPro S protein was conducted using SDS-PAGE analysis (4–12% NuPage™ SDS gel, ThermoFisher, Victoria, Australia) and size-exclusion chromatography (SEC) on a Superose 6 Increase 10/300 GL column (Cytiva, Marlborough, MA, USA) with Gel Filtration Calibration Kits containing a mixture of proteins (thyroglobulin, ferritin, aldolase, conalbumin, ovalbumin, carbonic anhydrase, Cytiva, Marlborough, MA, USA) as standards. The accuracy of the antigenic structure of HexaPro S protein was probed by indirect enzyme-linked immunosorbent assay (ELISA) using a panel of S-specific monoclonal antibodies (1047, 2M-10B11, CR3022, S309, hACE2, 2-17, 1-22, mAb 2.8, and mAb 18C2) [47,48,49,50,51,52].

### 2.3. Transmission Electron Microscopy (TEM)

Particle imaging was captured using a JEM-1010 transmission electron microscopy (TEM; HT7700 Exalens, HITACHI Ltd., JEOL Ltd., Tokyo, Japan) operated at 120 kV, using negative staining. Purified SARS-CoV-2 HexaPro S protein (~10 µg/mL in phosphate-buffered saline (PBS)) was applied to glow-discharged carbon–copper 400-mesh grids (ProSciTech, Queensland, Australia) for 2 min before washing three times with water. The grids were negative stained with 1% uranyl acetate and air-dried before imaging at 30 k magnification.

### 2.4. High-Density Microarray Patch (HD-MAP) Coating and Application

High-density microarray patches (HD-MAPs; Vaxxas Pty Ltd., Brisbane, Australia) were produced by injection molding medical-grade synthetic polymer. Each patch encompassed 5000 projections/cm^2^ microprojection arrays, with a tip diameter of 25 µm and a length of 250 µm for each. Prior to vaccine coating, these HD-MAPs were cleaned with oxygen plasma treatment (for 5 min at 30 W) at the Queensland Node of the Australian National Fabrication Facility (ANFF-Q).

A 21 µL of vaccine formulation, comprised of 2 µg of SARS-CoV-2 HexaPro S protein and excipients (0.25% human serum albumin (HSA) and 0.75% methylcellulose) mixed with or without 3 µg adjuvant QS-21 (Desert King International, San Diego, CA, USA), was pipetted to the patch surface. The solution was then dried using the previously reported, sterile-filtered nitrogen gas stream [42]. Before the HD-MAP application, the vaccination site of the mice was primed by removing the fur via shaving and depilatory cream. Vaccine-coated HD-MAPs were then applied to the flank of mice at a velocity of 18–20 m/s using a custom applicator. The patch was then removed from the skin after 2 min.

### 2.5. Vaccine Delivery Efficiency Using HD-MAP

The delivery efficiency of HD-MAP vaccines was analyzed using a capture ELISA. Nunc MaxiSorp ELISA plates (ThermoFisher, Victoria, Australia) were coated with mAb DH1047 [53] at 10 µg/mL in 50 µL of PBS and incubated at 4 °C overnight. The next day, plates were blocked for 30 min with 150 µL/well of 1x blocking buffer (5% KPL milk diluent/blocking solution concentrate; SeraCare, Milford, MA, USA). Vaccine-coated HD-MAPs (5 patches per vaccine candidates) that were delivered into the skin of mice or non-delivered control patches were eluted for 30 min in blocking buffer in the wells of a 24-well plate with plates shaking at 125 rpm. The samples were then serially diluted, added to the DH1047-coated ELISA plate, and incubated at 37 °C for 1 h before washing with PBS containing 0.05% Tween 20. Captured spike was detected using an in-house generated mAb 6A11 that was conjugated to horseradish peroxidase (HRP) before the addition of tetramethylbenzidine one component HRP microwell (TMBW; Sigma-Aldrich, St. Louis, MO, USA) substrate. The reaction proceeded for 5 min at room temperature (RT) before adding 1 M phosphoric acid to stop the reaction. Absorbance was read immediately at 450 nm using a Varioskan LUX Microplate reader (ThermoFisher, Victoria, Australia). Vaccine delivery efficiency was assessed by comparing the remaining SARS-CoV-2 HexaPro S protein delivered HD-MAPs to undelivered HD-MAPs.

### 2.6. Immunization in an In Vivo Model

Forty-eight naïve 6–8-week-old female BALB/c mice were purchased from Animal Resources Centre (Perth, Australia) and housed in the Australian Institute for Bioengineering and Nanotechnology Animal Facility (Queensland, Australia). The mice were acclimatized for 7 days before experimentation began. The mice (*n* = 8 per vaccine formulation) were given 2 µg of SARS-CoV-2 HexaPro S protein with or without the saponin-based adjuvant QS-21 (Desert King International, USA) via HD-MAP application or intradermal injection (i.d.). Each animal received a boost of the same dose on day 21. Negative control mice received vaccine vehicle (excipient) only.

### 2.7. Collection of Serum and Bronchoalveolar Lavage (BAL)

Serum samples were collected on days 20 and 42 post-vaccination to measure antigen-specific IgG antibody titers. Blood was collected via tail tip (on day 20) and heart puncture (day 42, after CO_2_ euthanasia). The blood samples were allowed to coagulate overnight at 4 °C before centrifugation at 10,000× *g* for 10 min at 4 °C. Serum was collected from the supernatant and stored at −20 °C until further analysis.

A bronchoalveolar lavage (BAL) was also performed at the time of cardiac puncture on day 42. The BAL fluid was centrifuged (1000× *g* for 5 min) to remove debris, and the supernatant was harvested and stored at −80 °C until further analysis.

### 2.8. Antibody Titer Detection by ELISA

Antigen-specific antibody IgG titers from serum were detected using ELISA, as previously described [42]. A 96-well plate was coated with 2 µg/mL of antigen (SARS-CoV-2 HexaPro S protein, HexaPro S protein from ancestral, Alpha or Delta variants) in PBS. Plates were blocked as before, and five-fold serial dilutions of serum or BAL samples were added to the plates. Blocking buffer only and monoclonal antibody CR3022 [54,55] were used as negative and positive controls, respectively. The plates were incubated for 1 h at 37 °C. Serum binding was detected using an HRP-linked goat anti-mouse secondary antibody and tetramethylbenzidine (TMB) substrate. Binding curves were analyzed using GraphPad Prism software (Version 9.0) with antibody titers presented as the serum value giving 50% of the maximum absorbance reading (EC50).

### 2.9. Virus Neutralization by Serum and BALs

#### 2.9.1. Virus Preparations

Virus isolates of SARS-CoV-2 recovered from nasopharyngeal aspirates of infected individuals and passaged twice on VeroE6 cells were provided by the Queensland Health Forensic and Scientific Services, Queensland Department of Health (Queensland, Australia). Virus isolates used in this study include: the early (ancestral) Australian isolate (i) hCoV-19/Australia/QLD02/2020 (GISAID Accession ID: EPI_ISL_407896, collected on the 30 January 2020); (ii) an isolate of the B.1.1.7 lineage, hCoV-19/Australia/QLD1517/2021 (referred to as the Alpha variant; GISAID Accession ID: EPI_ISL_944644: collected on 6 January 2021); (iii) an isolate of the B.1.617 lineage, hCoV-19/Australia/QLD1893C/2021 (referred to as Delta variant; GISAID Accession ID: EPI_ISL_2433928; collected on 4 May 2021). Virus isolates were further propagated on VeroE6 cells and stocks stored at −80 °C. Virus titer was determined by immunoplaque assay (IPA) as previously described [56].

#### 2.9.2. Plaque Reduction Neutralization Test (PRNT)

The neutralization activity of serum and BAL fluid against SARS-CoV-2 was evaluated using an established plaque reduction neutralization test (PRNT) [56]. Heat-inactivated samples were serially diluted in Gibco Dulbecco’s Modified Eagle Medium (DMEM; supplemented with 2% FBS and penicillin/streptomycin) before the addition of virus. The sample–virus mixture was incubated for 30 min at 37 °C and then added to confluent VeroE6 monolayers in 96-well plates. The infection process was conducted for 1 h at 37 °C before adding the overlay (1% carboxymethylcellulose, 5% fetal bovine serum (FBS)), and penicillin/streptomycin in Medium 199 (M199; ThermoFisher, Australia). Cells were then fixed with 80% acetone in PBS after 14–16 h post-infection. The plates were allowed to dry before the formed plaques were stained with SARS-CoV-2 S-specific mAbs (CR3022 or S309) and IRDye 800CW-conjugated goat anti-human secondary antibodies. Plates were scanned using an Odyssey CLX imaging system (LI-COR, Bad Homburg v. d. Höhe, Germany) to visualize the immunofluorescence plaques. Plaque numbers were counted using Viridot [57].

### 2.10. Ethics Statement

This study was performed according to strict regulations from the National Health and Medical Research Council of Australia (Australian Code of Practice for the Care and Use of Animals for Scientific Purposes, 8th edition 2013). The University of Queensland Animal Ethics Committees (AEC) approved all animal procedures and protocols, AEC Approval Number: SCMB/322/19/AIBN. The work with SARS-CoV-2 was performed under the University of Queensland Institutional Biosafety Committee (UQ IBC) approval number: IBC/390B/SCMB2020, IBC/1301/SCMB/2020, IBC/376B/SBMS/2020 and IBC/447B/SCMB/2021.

### 2.11. Statistical Analysis

GraphPad Prism 9.0 (GraphPad Software, Inc., San Diego, CA, USA) software was used for statistical analysis and generation of graphs and figures. As described in the figure legends, data are presented as mean with standard deviation (SD) or standard error of the mean (SEM). Statistical significance was determined via one-way ANOVA with Tukey’s multiple comparison test. Data were considered significantly different at (*) *p <* 0.05, (**) *p <* 0.01, (***) *p <* 0.001, and (****) *p <* 0.0001 among the studied group.

## 3. Results

### 3.1. Characterization of SARS-CoV-2 HexaPro S Protein

**Figure 1:**
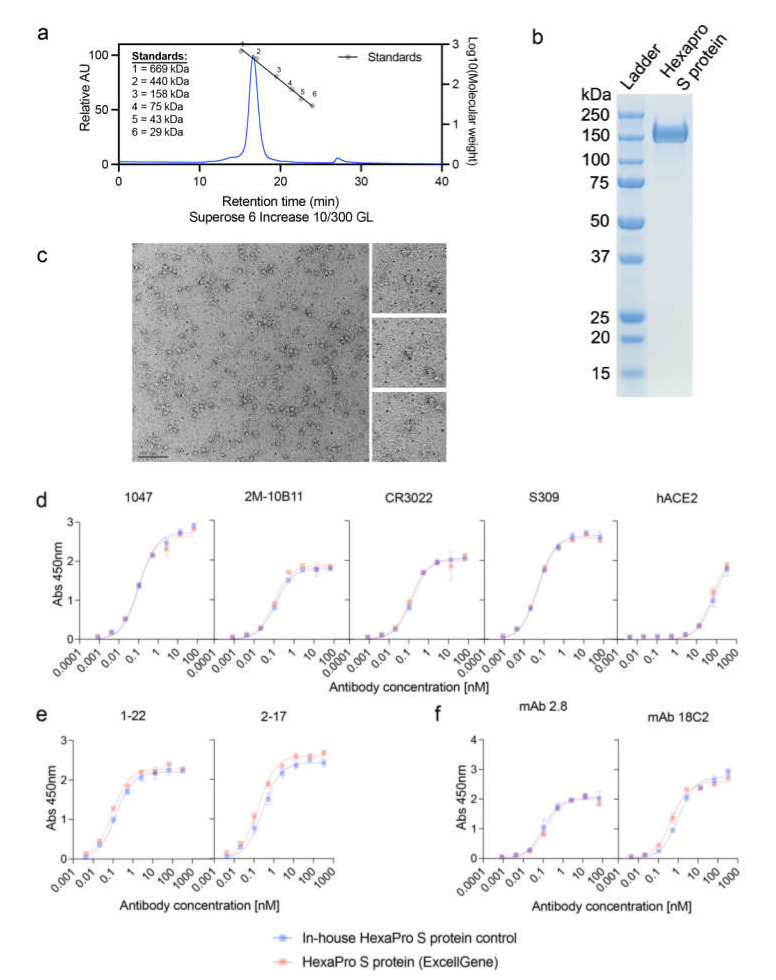
Characterization of the SARS CoV-2 HexaPro S protein. (**a**) Analysis of HexaPro S protein on size-exclusion chromatography (SEC), with a single peak at 16.6 min and purity of 98%. A mixture of thyroglobulin (669 kDa), ferritin (440 kDa), aldolase (158 kDa), conalbumin (75 kDa), ovalbumin (43 kDa), and carbonic anhydrase (29 kDa) proteins were used as standards. (**b**) SDS-PAGE gel showing a band for the HexaPro S protein. (**c**) Negative-stain electron microscopy (Bar = 100 nm) of the HexaPro S protein. Antibody binding to (**d**) RBD (1047, 2M-10B11, CR3022, S309, and hACE2), (**e**) N-terminal domain (NTD; 2-17 and 1-22), and (**f**) S2 subunit (mAb 2.8 and mAb 18C2).

Initial characterization of the commercially sourced SARS-CoV-2 HexaPro S protein (hereafter known as HexaPro S protein; ExcellGene, Switzerland) was carried out to confirm the purity along with the structural and antigenic authenticity of the protein (Figure 1). To confirm the oligomeric nature of the supplied HexaPro, we performed analytical SEC and SDS-PAGE analysis. The SEC analysis confirmed the presence of a single 440 kDa species, which corresponds to the molecular weight of the trimeric HexaPro structure (Figure 1a). Protein separation under denaturing conditions on an SDS-PAGE observed a single 150–160 kDa band (Figure 1b). Having confirmed the molecular weight and purity of the protein, TEM analysis of the protein revealed the classic spike “kite-like” structure (Figure 1c), confirming the structural integrity of the protein [58].

Following confirmation of the overall structure of the HexaPro S protein, the antigenic authenticity of the protein was investigated by indirect ELISA. A panel of anti-spike antibodies directed against multiple domains of the protein, RBD (Figure 1d), N-terminal (Figure 1e), and S2 domains (Figure 1f), were evaluated. The binding profile of hACE2 was also performed using the cellular receptor of ACE2, which is tagged with a monomeric human Fc to facilitate purification and detection using anti-human secondary antibodies. Antibody binding profiles were evaluated against both the commercially sourced HexaPro and an in-house HexaPro S protein control that was previously validated. As seen in Figure 1d–f, the binding profiles of all the antibodies to both HexaPro S proteins were identical, indicating that the HexaPro S protein is antigenically authentic. Having confirmed that the commercially sourced HexaPro S protein was both antigenically and structurally authentic, we proceeded with HD-MAP coating and immunization studies.

### 3.2. Vaccine Delivery via HD-MAPs 

**Figure 2:**
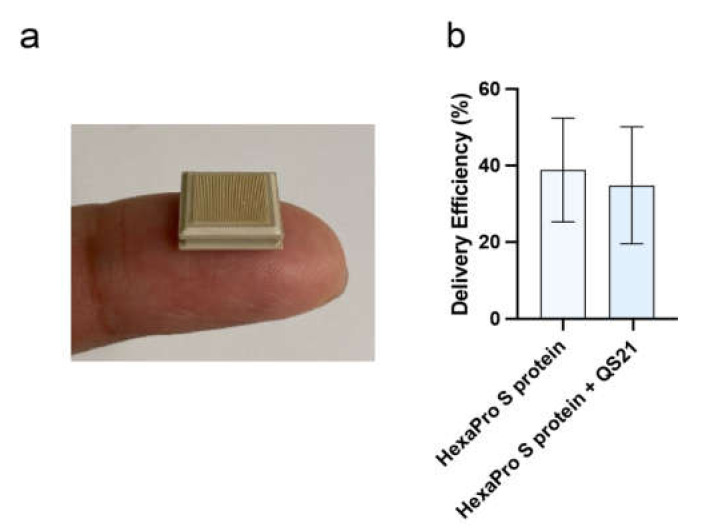
HexaPro S protein-based vaccine and its application using HD-MAP. (**a**) HD-MAPs containing 5000 solid polymer microprojection arrays to deliver vaccine into the cutaneous layer of the skin. (**b**) Delivery efficiency of antigen into the skin using HD-MAPs coated with SARS CoV-2 HexaPro S protein and QS-21-adjuvanted SARS CoV-2 HexaPro S protein (*n* = 5, each) was measured by comparing the remaining protein from the delivered HD-MAPs to undelivered HD-MAPs using capture ELISA.

Accurate determination of the deposition of vaccine payload by the HD-MAP into the skin is essential to ensure appropriate dosing. Vaccine delivery efficiency assays were performed to determine the conditions required to deliver 2 µg of HexaPro to the skin via the HD-MAP. The HexaPro S protein was formulated with 0.25% HSA and 0.75% methylcellulose with and without 3 µg of the adjuvant QS-21 and dry-coated onto the microprojection of the HD-MAP using a nitrogen gas jet (Figure 2a). To quantify the amount of vaccine delivered, a spike-specific ELISA was performed to measure the vaccine remaining on the microprojections following application. Using this approach, we determined delivery to be 39% and 35% for the HexaPro S protein formulated with and without QS-21, respectively (Figure 2b).

### 3.3. Immune Responses Following HD-MAP Vaccination

**Figure 3:**
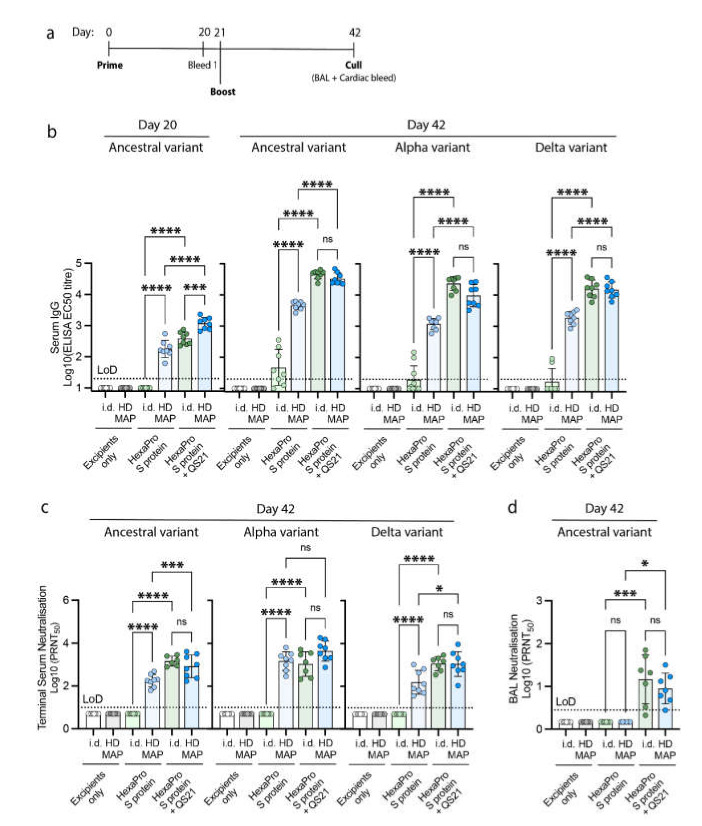
Immune responses in BALB/c mice following the vaccination with excipients (negative control), HexaPro S protein, and HexaPro S protein + QS-21 via intradermal injection (i.d.) or HD-MAP application (*n* = 8, each), (**a**) vaccination schedule. Serum was collected after primary immunization and first boost (on day 20 and 42, respectively) and analyzed for (**b**) serum IgG antibody titers against HexaPro S protein and HexaPro S protein derived from Alpha and Delta variants by ELISA. Serum and bronchoalveolar lavage (BAL) collected on day 42 were analyzed for (**c**) serum virus neutralization by plaque reduction neutralization test (PRNT) against the parental SARS-CoV-2 isolate, an Alpha variant, and a Delta variant, and (**d**) BAL virus neutralization by PRNT against ancestral SARS-CoV-2 variant, respectively. Each point represents an individual biological replicate (mouse) performed on a single ELISA assay; bars represent the average antigen-specific IgG antibody titers (EC50); error bars represent the SD; the LoD line represents the assay limit of detection. Statistical analysis was performed using one-way ANOVA with Tukey’s multiple comparison *post hoc* test ((*) *p <* 0.05, (***) *p <* 0.001, (****) *p <* 0.0001 and non-significant (ns)).

Following the successful coating and determination of vaccine delivery, we sought to evaluate the immunogenicity of HexaPro S protein with or without the adjuvant QS-21 via either HD-MAP application or i.d. injection. Female BALB/c mice (*n* = 8 per vaccine formulation) were vaccinated with two doses, each 21 days apart, of 2 µg of HexaPro S protein with or without 3 µg of QS-21. Serum samples were collected on day 20 and 42 post first vaccination and analyzed for total IgG and virus-neutralizing titers. Excipients only HD-MAPs were also included as controls. After the first dose, no detectable levels of IgG antibodies were observed for the HexaPro S protein i.d. group (Figure 3b). Although S-specific antibodies were eventually raised in the HexaPro S protein i.d. groups (after second dose), IgG levels from the corresponding HD-MAP-delivered HexaPro S protein were both significantly higher than those from the i.d. vaccinated counterparts (HexaPro S protein HD-MAP: *p <* 0.0001; HexaPro S protein + QS-21 HD-MAP: *p =* 0.0007; Figure 3b). Following the second immunization, sera obtained were evaluated against the ancestral, Alpha, and Delta variant HexaPro S proteins. Although only a subset of mice seroconverted from the HexaPro S protein i.d. group, the remaining groups observed a 1 log increase in antibody titers after the second dose. IgG titers from the HexaPro S protein + QS-21 groups were observed to be significantly higher than their unadjuvanted counterparts when evaluated against the three variants regardless of delivery method (*p <* 0.0001 (for all three variants), Figure 3b). Promisingly, the antibodies generated following HexaPro S protein vaccination were highly effective in recognizing HexaPro S proteins from both Alpha and Delta variants (Figure 3b).

To confirm that the IgG elicited from the HexaPro S protein-vaccinated mice was functional, we proceeded to analyze the sera for their ability to neutralize the virus. Reflecting what was observed for the IgG titers, mice vaccinated with HexaPro S protein by i.d. injection had no detectable neutralizing antibodies across the three variants (Figure 3b). No significant differences were observed between the different delivery methods for the adjuvanted group, while the opposite was observed for the unadjuvanted groups. Mice receiving the HexaPro S protein delivered via the HD-MAP had significantly higher virus-neutralizing antibody levels as compared to the i.d. injected group (*p <* 0.0001 (for all three variants); Figure 3c). Interestingly, no significant differences were observed when evaluated against the Alpha variant of concern between antibody levels for the HexaPro S protein with and without QS-21 when delivered via HD-MAP (Figure 3c).

The mucosal surface is an important site of immunity for respiratory pathogens such as SARS-CoV-2 as it could offer a first line of defense against virus infection. To assess the functionality of antibodies at a mucosal level, bronchoalveolar lavage (BAL) was performed in the lungs of the mice. PRNTs were analyzed using the samples obtained from the BAL. Despite observing IgG and neutralizing antibodies from blood sera obtained from mice vaccinated with HexaPro S protein only, no BAL neutralizing antibodies were elicited regardless of the vaccine delivery method (Figure 3d). BAL neutralizing antibodies were observed for the adjuvanted group; however, no significant differences were observed between the delivery method (Figure 3d).

## 4. Discussion

The ongoing COVID-19 pandemic caused by the SARS-CoV-2 virus remains a major global health burden. Although multiple effective vaccines are available for emergency use, many challenges are faced in the rollout of these vaccines due to complex cold chain logistics. Therefore, there is an urgent need for thermostable and easy-to-administer vaccines, enabling broader distribution and global access. We have previously described a thermostable COVID-19 HD-MAP vaccine candidate, delivering the HexaPro S protein [42]. Protein-based subunit vaccines are simple to manufacture and have historically excellent safety profiles [59]. The potent antibody response elicited was found to effectively protect from SARS-CoV-2 challenge and neutralized a wide variety of viral isolates, including the Alpha and Beta variants of concern [42]. Following the completion of our previous study, the Delta variant has emerged as the dominant variant of concern globally. Here we evaluated the performance of commercially sourced HexaPro S protein (ExcellGene, Switzerland) and its ability to elicit neutralizing antibody responses against the Delta variant in mice.

Through the various initial characterizations and analyses, HexaPro S protein was observed to be structurally and antigenically authentic. HexaPro S protein was also observed to have the same antibody binding profile as our in-house HexaPro S protein control, which was previously validated and offered protection from virus challenge after a single adjuvanted dose delivered by HD-MAP [42]. The effective presentation of the antigenic protein is critical for recognition by immune cells and the subsequent immune response elicited [60]. Upon confirmation of HexaPro S protein’s structural and antigenic authenticity, mice were immunized with two doses of vaccine via HD-MAP application or i.d. injection.

The superior immune response elicited when vaccinated using the HD-MAP was again observed in this study. Unadjuvanted HexaPro S protein delivered via the HD-MAP resulted in significantly higher IgG and virus-neutralizing antibodies as compared to the i.d. injected group, with similar levels to the HexaPro S protein + QS-21 i.d. group after one dose. These findings are consistent with previous studies, with HD-MAP eliciting an enhanced immune response as compared to the traditional needle and syringe method [41,42,61,62,63,64,65,66,67]. The combination of the vaccine payload being delivered to the immune-rich layers of the skin and localized cell death triggered by the mechanical stress upon HD-MAP application contributes to the enhanced immune response observed [39,40]. This is likely a reflection of the tumor necrosis factor and nuclear factor-kB signaling pathways triggered by the vaccine-induced adaptive immune responses [40]. The absence of neutralizing antibodies from the HexaPro S protein delivered via the i.d. injection route again supports the evidence that the HD-MAP delivery method is superior to i.d. injection in producing enhanced immune responses. This is especially true with subunit protein vaccines as they are weakly immunogenic and often require some form of adjuvant to help boost the immune response elicited [68].

The adjuvating effect of QS-21 was observed after both one and two doses in this study. After two doses, IgG levels in all groups with QS-21 were raised to a similar level. The self-adjuvanting effect of HD-MAP was overshadowed by the strong adjuvanting effect of QS-21 in the vaccine formulation. Hence, no difference was observed between the intradermal and the HD-MAP delivery of QS-21-adjuvanted HexaPro S protein. QS-21 has been observed to broaden immune responses and enhance cell-mediated immunity [69]. This was observed in this study, with both HD-MAP and i.d. groups containing QS-21 able to neutralize not only the ancestral virus but also the Alpha and Delta variants of SARS-CoV-2. Virus-neutralizing antibody titers were also observed to be raised to similar levels across the three variants, suggesting that the broad immune response elicited by HexaPro S protein combined with QS-21 is able to overcome the mutations in the Alpha and Delta variants. This highlights the importance of adjuvants when delivering subunit vaccines. The mutations in the Alpha and Delta variants of concern have been shown to result in immune evasion of serum induced by vaccination or previous infection, though protection from severe disease remains high [70,71]. In November 2021, the Omicron variant emerged and was rapidly declared by the WHO as a variant of concern. The heavily mutated HexaPro S protein impacted the virus’s infectivity and transmission [72,73,74]. The Omicron variant has also been observed to evade neutralization by antibodies obtained from convalescent patients or those previously vaccinated with the AstraZeneca and Pfizer vaccines at a higher efficacy than the Delta variant [75]. Therefore, a follow-up study to analyze the efficacy of this vaccine candidate against the Omicron variant is necessary. It is imperative to evaluate the effectiveness of protein-based vaccines against novel variants of concern to control the pandemic and reduce the incidence and severity of COVID-19 infections.

## 5. Conclusions

In conclusion, this study investigated commercially sourced HexaPro S protein as a vaccine candidate. The findings observed that it is structurally and antigenically authentic to the in-house HexaPro S protein that was previously validated. Virus-neutralizing antibodies produced by mice vaccinated with two doses of QS-21-adjuvanted HexaPro delivered by HD-MAP were able to neutralize emerging variants of SARS-CoV-2 at a similar level to the ancestral variant. While its efficacy against newly emerged variants of concern (e.g., Omicron) has yet to be evaluated, these findings present it as a potentially effective vaccine alternative capable of mitigating the impacts of emerging viral variants. Moreover, our results provide a proof-of-principle for vaccination against SARS-CoV-2 using HD-MAP-delivered HexaPro S protein. This novel vaccine concept could help to ensure global access, availability, and affordability of COVID-19 vaccines by abolishing the need for cold storage and the need for specialized medical personnel for administration of the vaccine. These unique properties of this vaccine approach are very important to reach hard to reach populations in low- and middle-income countries.

## Data Availability

Not applicable.
